# 646 Determinants of Access to Care Among Pediatric Patients with Post Burn Contractures

**DOI:** 10.1093/jbcr/iraf019.275

**Published:** 2025-04-01

**Authors:** Alice Umutoni, Blaise Habineza, Jonathan Ayeyi Nuamah, Shirley Dadson, Mohammed Nurudin Osman, Ebenisha Choonya Majata, Sarah Okojie, Ulrick Kanmounye, Faustin Ntirenganya Ntirenganya, Justin Bayisenga, Charles Furaha

**Affiliations:** Operation Smile International; Kigali University Teaching Hospital (CHUK); University of Ghana Medical School; University of Ghana Medical School; University of Ghana Medical School; Copperbelt University, Michael Chilufya Sata School of Medicine; Lagos State University Teaching Hospital; University of Ghana Medical School; University of Rwanda; Kigali University Teaching Hospital (CHUK); Rwanda Military Referral and Teaching Hospital

## Abstract

**Introduction:**

Post-burn contractures (PBC) are among the major complications of burn injuries yet knowledge about the factors affecting healthcare access among patients with PBC is limited. This study aims to assess the barriers and facilitators to care among pediatric post-burn contracture patients.

**Methods:**

A cross-sectional study including patients admitted for PBC between April -August 2022. A pre-established questionnaire was administered to assess socio-demographic characteristics and probable factors determining access to care. Logistic regression was used to assess model fit of missed follow-up appointments against independent variables. The area under the curve and goodness of fit were computed. A p-value < 0.05 was considered significant.

**Results:**

A total of 390 pediatric patients have been included in this study. The majority of patients were female (53.5%) with a median age of 7 years. Most patients had health insurance (97.5%) and belonged to Ubudehe categories 2 (48.8%) or 3 (39.3%). Scalds were the most common burn type (60.4%). The proximal interphalangeal joint (PIP) and elbow were the most frequently affected areas. The most common post-burn contracture type was MASCCA B (48.2%). Regular exercise in the affected region was the most reported facilitator for attending follow-up appointments (76.4%). Lack of money was the most prevalent barrier among those who missed appointments (100%). Patients from lower socio-economic backgrounds (Ubudehe 1) had access to fewer facilitators and reported more geographical barriers. Attending physiotherapy sessions post-discharge significantly reduced the odds of missing follow-up appointments (OR = 0.41, p = 0.02).

**Conclusions:**

Geographical, educational, and financial barriers emerged as the most important issues to access burn care. We recommend increasing burn prevention measures, improving burn care at lower levels of the Rwanda Health system, enhancing patients’ and families’ education, regular exercise, and better access to occupational therapy.

**Applicability of Research to Practice:**

These results highlight the need for interventions that promote physiotherapy adherence and address financial limitations to improve post-burn contracture care, decentralization of particularly for those from lower socioeconomic backgrounds.

**Funding for the Study:**

N/A

